# GRANULOMA ANNULARE - REMISSION WITH PUVASOL

**DOI:** 10.4103/0019-5154.60363

**Published:** 2010

**Authors:** Chembolli Lakshmi, C R Srinivas

**Affiliations:** *From the Department of Dermatology, PSG Institute of Medical Sciences and Research, India.*

**Keywords:** *Bath-PUVA*, *granuloma annulare*, *solarium*

## Abstract

A 64-year-old man with generalized papular granuloma annulare of four years duration with frequent relapses and remissions presented with persistent lesions while on 15 mg prednisolone and achieved complete remission with PUVASOL therapy using solarium.

## Introduction

Granuloma annulare is a benign dermatosis of unknown aetiology. Generalized lesions occur in approximately 15% of patients with granuloma annulare. Generalized granuloma annulare has been defined as lesions occurring on at least the trunk and either upper or lower, or both, extremities.

The disease is very persistent and resistant to treatment. Various treatment options include PUVA therapy, dapsone and isotretinoin. We report a 64-year-old man with generalized granuloma annulare who achieved remission with PUVASOL using the solarium

## Case Report

**Figure 1:**
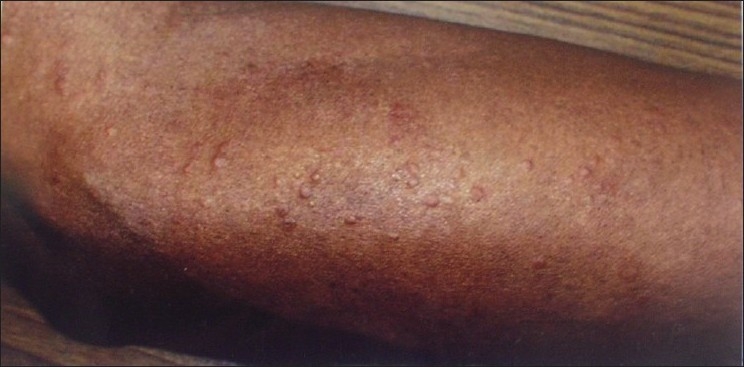
Erythematous papules over forearm

A 64-year-old male presented with generalized pruritic, erythematous papules which recurred and remitted with treatment for the past four years. The lesions healed without leaving scars [Figure 1]. The lesions persisted initially for two months but during the present episode, the lesions had persisted for more than six months, in spite of treatment with systemic steroids. In addition, he had polyarthritis and recurrent chest infections in the past.

Examination revealed multiple erythematous papules distributed over the arms, legs, both sides of the trunk and palms sparing the face, neck, gluteal region and soles. There was no tenderness or joint swelling. We considered granuloma annulare, multicentric reticulohistiocytosis, eruptive xanthoma, papular sarcoidosis and cutaneous tuberculosis and the patient was investigated.

**Figure 2:**
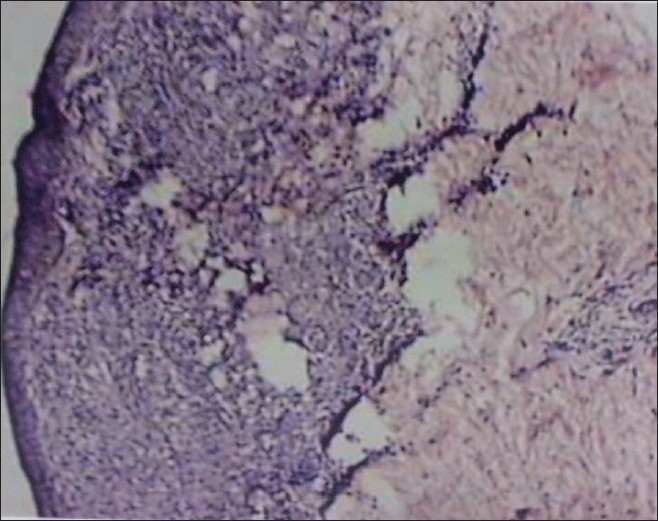
Granulomas in the papillary dermis without apparent necrobiosis

All routine investigations were normal except for a raised ESR. The lipid profile and liver function tests, serum calcium, serum ACE (angiotensin converting enzyme) were normal. Chest radiograph showed features of healed tuberculosis and the Mantoux test was negative. Histopathology revealed collections of epithelioid histiocytes and lymphocytes forming granulomas in the papillary dermis without apparent necrobiosis [Figure 2]. Stain for reticulin was negative. He was diagnosed with diffuse granuloma annulare after clinico-histopathological correlation.

Bath-PUVA with sun exposure using the solarium was administered initially twice a week along with prednisolone 15 mg, which was tapered to 5 mg. Within a month and a half, he attained complete remission. Weekly PUVASOL therapy using the solarium and 5 mg prednisolone was continued and he continued to be free from lesions. The patient was irregular in coming for treatment and four months later the lesions recurred. PUVASOL therapy was re-instituted.

## Discussion

Granuloma annulare is a benign, usually self-limited dermatosis of unknown cause. It is widely believed to be an immunologically mediated reaction in which inflammation surrounds blood vessels and alters collagen and elastic tissues.

The clinical variants include localized, generalized, subcutaneous, perforating, patch, arcuate dermal erythema and actinic granuloma.[1] A follicular pustular variant has also been described.[2] About 15% of all patients present with the generalized variety. The lesions are symmetrical with the face, palm, soles and mucous membranes being involved only rarely. A negative Mantoux and lack of response to anti-tuberculous treatment ruled out active tuberculosis. Normal serum calcium and ACE levels, along with histopathology suggestive of diffuse granuloma annulare, and a negative reticulin stain ruled out sarcoidosis. Diffuse granuloma annulare resembles lichen nitidus histopathologically, but lacks the acanthotic downgrowths of epidermis at the periphery of the lesion.[2] Although necrobiosis was not seen in this case, necrobiosis is inconspicuous in the diffuse variant.[2] The first line therapies include PUVA therapy, dapsone and isotretinoin. Psoralen plus ultraviolet A (PUVA) therapy has been reported to improve and even cure granuloma annulare[3,4] and is the treatment of choice for a patient with widespread diseases.

The mechanism of action of oral PUVA in granuloma annulare is unclear; however, the induction of apoptosis in pathogenetically relevant cells is a possibility.[5] Topical application of psoralens followed by ultraviolet- A radiation is effective.[6] To minimize the side effects of oral PUVA therapy, we administered bath water psoralens soaks by standard protocol following which the patient was exposed to ultraviolet rays from sunlight (PUVASOL) using the solarium.[7] There was complete clearance of lesions within one-and-a-half months. Since the patient was irregular in coming for treatment, he developed new lesions after four months. Lesions remitted with re-institution of PUVASOL therapy. Several reports have also reported recurrence with cessation of therapy.[3,4] We plan to maintain the patient on PUVASOL to achieve a longstanding remission.
